# The Translation and Validation of the Children’s Health Internet Research Parental Inventory (CHIRPI) in Greek: A Crucial Tool for Evaluating Parent Internet Use for Children’s Health Information

**DOI:** 10.3390/children12081049

**Published:** 2025-08-10

**Authors:** Danai Maria Psoma, Stavroula Ilia, George Briassoulis, Antonia Barke, Bettina K. Doering, Aggeliki Xirorafa, George Notas

**Affiliations:** 1 Postgraduate Program “Emergency and Intensive Care in Children, Adolescents and Young Adults”, School of Medicine, University of Crete, 71003 Heraklion, Greece; ddk296@edu.hmu.gr (D.M.P.); stavroula.ilia@uoc.gr (S.I.); briasoug@uoc.gr (G.B.); ddk297@edu.hmu.gr (A.X.); 2 Pediatric Intensive Care Unit, University Hospital, School of Medicine, University of Crete, 71003 Heraklion, Greece; 3 Institute of Psychology, University of Duisburg-Essen, 45141 Essen, Germany; antonia.barke@uni-due.de; 4 Department of Psychology, Kiel University, 24098 Kiel, Germany; doering@psychologie.uni-kiel.de; 5 Department of Emergency Medicine, University Hospital, School of Medicine, University of Crete, 71003 Heraklion, Greece

**Keywords:** CHIRPI, parental behavior, online health information, psychometric validation, Greek translation, pediatric information-seeking

## Abstract

The internet is now the primary mode of information exchange worldwide. Online health information-seeking behavior (e-HISB) has become a prevalent practice, especially among parents concerned with their children’s health, creating both opportunities and risks. **Objective:** The present study aims to translate and culturally adapt the CHIRPI questionnaire into Greek and conduct a comprehensive psychometric validation, including analyses of internal consistency, test–retest reliability (temporal stability), and inter-rater reliability. The adapted tool is further pilot-tested for its utility in measuring parental internet use concerning child health information. **Methods**: The translation, validation, and pilot study of the questionnaire were conducted in accordance with internationally recommended procedures. CHIRPI was translated into Greek using forward–backward translation and was culturally adapted. A pilot sample of 105 parents (children aged 0–10) participated. The majority of participants were mothers (66.7%), aged 31–40 years, residing in urban areas, and they held tertiary or postgraduate degrees. Internal consistency was measured with Cronbach’s alpha, test–retest reliability with the ICC, and inter-rater reliability with the kappa coefficient. Item responses were also analyzed in relation to demographic factors. **Results**: The CHIRPI Greek version demonstrated excellent internal consistency (Cronbach’s α = 0.91; all subscales had α values greater than 0.70). Test–retest reliability (ICC = 0.632–1.000) and inter-rater reliability (kappa = 0.615–1.000) indicated moderate to excellent agreement. The scale showed satisfactory psychometric properties, supporting its use in Greek populations. Higher education was linked to more frequent health-related internet searches and increased distress (*p* < 0.001). **Conclusions**: The CHIRPI Greek version is a valid and reliable tool for assessing parental online health information-seeking behavior related to children’s health among Greek-speaking populations. As the first standardized tool in Greek, it fills a critical methodological gap in eHealth research.

## 1. Introduction

The search for information regarding health issues is essential for assessing, maintaining, and changing one’s health and health behaviors [1,2]. Medical information can be gathered from formal sources, such as healthcare providers, as well as informal sources like family or friends or other media sources [3]. The internet has emerged as an alternative option in recent years, becoming the primary means of information exchange worldwide. Unexpectedly, it has also become a significant source of health-related information (HRI) [4,5]. However, the role of the internet in seeking information related to health issues comes with challenges. While it serves a purpose by providing information that was not readily accessible in the past, it also poses the risk of misinformation, underscoring the need for further research and understanding [6]. This highlights the importance of caution and the need for additional research in this field.

Parents who are not healthcare professionals feel that the internet is a powerful tool for obtaining information about their children’s health [7]. They believe that the internet provides reliable and up-to-date health information and, therefore, the necessary knowledge to make informed decisions about their children’s health. Information from the web can significantly impact a child’s health, as it may determine whether and when the family will seek medical assistance [8]. Parents may also turn to the web when dissatisfied with their doctor’s information or to look for a different perspective [2]. However, being aware of the potential downsides to relying on online health information, including significant risks of misinformation and cyberchondria, is crucial [9,10].

Several tools have been developed to evaluate this phenomenon and appraise its implications, including the eHealth Literacy Scale (eHEALS) [11] and the Cyberchondria Severity Scale (CSS) [12], but not all of them have been adequately validated. Despite their relevance, these tools were not specifically designed to assess parental behavior, unlike CHIRPI. A recent systematic review examined the characteristics of these tools, including completion methods, psychometric properties, reliability, validity, dimensions, and appropriateness for measuring parental children’s health information-seeking behavior. Most of the recently developed tools are brief and self-administered and display decent psychometric characteristics [11,12,13,14,15,16]. Among the evaluated tools, the Children’s Health Internet Research, Parental Inventory (CHIRPI) was found to be one of the most appropriate [17]. The original German version of CHIRPI demonstrated strong psychometric properties, including a Cronbach’s alpha of 0.89 across the total scale. To our knowledge, it has not yet been widely translated into other languages, highlighting the importance of the present Greek adaptation.

CHIRPI is an instrument reflecting current digital health behaviors and parental engagement with online content. It is brief and easy to complete, minimizing respondent burden and enhancing response rates, while it is self-administered, allowing for flexible application. Most importantly, CHIRPI is specifically designed to measure parental online health information-seeking behavior concerning their children’s health, making it highly aligned with the aims of the present study. Finally, it demonstrates strong psychometric properties, with proven reliability and validity, which is essential for ensuring the quality and interpretability of the collected data.

These characteristics collectively positioned CHIRPI as the most suitable tool for translation, cultural adaptation, and implementation in the context of Greek-speaking populations. Our study aimed to translate, validate, culturally adapt, and assess the psychometric properties of the CHIRPI questionnaire in the Greek language and specifically analyze its internal consistency, temporal stability, and inter-rater reliability. Additionally, we sought to assess the feasibility of using this questionnaire in Greek and to ensure that the translated tool is both practically applicable and methodologically sound for conducting research among Greek-speaking populations.

## 2. Materials and Methods

### 2.1. The CHIRPI Scale

The CHIRPI scale, developed in 2020 by Antonia Barke and Bettina Doering, can be completed by parents within approximately 5 to 10 min and uses questions answered via a Likert scale (1 = never to 5 = always) that indicate how often parents exhibit a specific behavior or experience a particular situation [17]. CHIRPI consists of 21 items, organized into 3 subscales of 7 items each, describing the cognitive, emotional, and behavioral aspects of parents’ online searches for information regarding their children’s health. The subscales are as follows: “Distress,” “Symptom Focus,” and “Implementing Advice”. Specifically, the subscale “Distress” measures the negative emotional consequences that parents experience when searching the internet for information related to their children’s health, such as anxiety, irritation, difficulty sleeping, and interference with their daily activities. Additionally, the subscale “Symptom Focus” captures the search behavior adopted by parents to find the causes or treatment for a presumed illness when any physical change occurs in their children. The subscale “Implementing Advice” measures the tendency of parents to apply advice based on online information, such as consulting their child’s doctor to prescribe a specific medication or undergo certain diagnostic procedures. Example items reflecting these dimensions include statements such as the following: “I find it hard to relax after searching online for health information concerning my child.” (Distress). “When my child displays any symptom, I search on the Internet for information about it.” (Symptom Focus). “I ask my child’s doctor to prescribe particular drugs that I have read about online.” (Implementing Advice).

### 2.2. Translation, Validation, and Cultural Adaptation

Prior to the translation process, the creators of CHIRPI, Antonia Barke and Bettina K. Doering, were contacted and informed via email about the purpose of this research. Written consent was requested from Antonia Barke for the translation and adaptation of the scale into the Greek language. Permission was granted electronically on 1 November 2022. The translation process of CHIRPI into Greek lasted less than one month, from 30 June to 18 July 2022. The translation process was based on the internationally accepted methodology, as outlined by the World Health Organization (WHO) [18]. Specifically, it involved the Forward and Blind Backward Translation of the tool, with the evaluation of the various versions and a pilot test to assess face validity. Regarding the Forward Translation, the CHIRPI tool was translated from English to Greek by two independent, bilingual translators with a high level of proficiency in English, who reside permanently in Greece and whose native language is Greek. The two translators created two separate printed translations, and following comparison, they were merged to create a final version of the translated questionnaire in Greek (1st reconciliation version). The translators worked closely with the research team (D.M.P., G.N., S.I., G.B.), who are medical professionals, to ensure the accurate rendering of medical terms. Also, the researchers (D.M.P., G.N.) discussed ambiguous or disputed items with the instrument developers as appropriate. No significant discrepancies arose regarding the final form of the translated Greek questionnaire. The researchers (D.M.P., G.N.) made the final decision on some minor discrepancies that existed in the final form of the translated Greek questionnaire. Thus, this final version was translated back into the original foreign language (backward translation), that is, English, by a bilingual individual whose native language is English, who permanently resides in Greece, and who was unfamiliar with the original version of the questionnaire. Finally, the backward translation was sent to the initial authors of the questionnaire for comments and feedback. The Greek version of the questionnaire was finalized after the review committee (D.M.P., G.N., S.I., G.B.) resolved all discrepancies. Appendix A presents the English and the Greek versions of the questionnaire, and the final format of the Greek questionnaire can be found in the Supplementary materials section.

The translated questionnaire was pre-evaluated in a small-scale sample of 12 participants, who were Greek-speaking parents of young children aged 0–10 years, selected through purposive sampling. Individual, qualitative semi-structured interviews were conducted to explore the participants’ understanding of all the questionnaire items. To ensure face validity, a cognitive debriefing process was implemented. Participants were asked to read each item aloud, paraphrase it in their own words, and comment on its clarity, relevance, and emotional tone. The evaluation aimed to identify misunderstandings, ambiguities, or challenges that participants might face while completing the questionnaire [19]. Items that were perceived as confusing or ambiguous would have been discussed by review committee with the original authors of CHIRPI and revised where necessary. However, no such difficulties were reported during this process, and therefore no changes were deemed necessary. Subsequently, to validate the questionnaire for the Greek-speaking population, a pilot study was conducted on a sample of 105 parents from the target population [20].

Validation was conducted by assessing reliability (evaluation of random error, precision, reproducibility, repeatability, and consistency) [21] and test–retest reliability. Internal consistency was examined using Cronbach’s alpha, and test–retest reliability was assessed through the intraclass correlation coefficient (ICC) after the second completion of the questionnaire by 100% of the participants one week later. For this test–retest reliability process, the order of the questions was changed to ensure that participants would not remember their responses from the first completion.

Demographic questions were added to the questionnaire regarding age, gender, education level, place of permanent residence, family health status, and whether the parents’ profession is related to any medical or paramedical profession.

### 2.3. Sample and Data Collection

As part of this study, the English version of the questionnaire was validated in Greek by 105 parents whose children were brought to the Pediatric Emergency Department of the University Hospital of Heraklion, Crete, Greece. Its implementation occurred from August 2022 to early January 2023. The researcher responsible for distributing the questionnaires asked the parents of children who had already been examined by the doctors to participate in the current study. The researcher informed them about the purpose of this research, briefly described the questionnaire, and assured them of their anonymity. Respondents were given a “quick response” code (QR code), which redirected the user to a consent form document. After giving consent, they were directed to a coded questionnaire hosted on a secure Google Form. The researcher supported the participants with the process if needed. Each questionnaire was assigned a unique identification number. Appropriate measures were taken to protect the anonymity of the subjects. To assess test–retest reliability, the researchers decided to send the questionnaire via email after seven (7) days to all parents who had completed the questionnaire (n = 105) without notifying them prior. The seven (7)-day interval was considered appropriate for the parents to respond to the questionnaire without forgetting their experience or recalling the answers they provided the first time they completed the questionnaire, thus avoiding memory bias. Additionally, the order of the questions was changed to ensure that participants would not remember their answers from the first completion of the questionnaire. A small number of parents required a follow-up email to be sent within seven (7) days, while five individuals who did not respond to this gentle reminder received a phone reminder one (1) week after the email was sent. Thus, the electronic method used in the second phase of completing the questionnaire, combined with phone reminders, ensured a complete response rate (100%) for the second phase of this study (retest).

### 2.4. Exclusion Criteria

The parents of children older than ten years, parents of children whose medical conditions had deteriorated to the extent that admission to a Pediatric Intensive Care Unit was required (to avoid potential stress-related bias), and parents who were healthcare professionals were excluded from this study.

### 2.5. Data Analysis

The data collected from the questionnaires during the initial test and the retest were coded and entered into a spreadsheet (Microsoft Office Excel). An analysis of the research data was conducted using IBM SPSS 28.0 software. The sample size needed was calculated to be 98 (sensitivity of 90%, specificity of 80%, α level of 0.05). A power analysis was conducted prior to data collection to estimate the required sample size. The analysis was based on the expected psychometric evaluation criteria and aimed at ensuring adequate statistical power for detecting moderate to high internal consistency (Cronbach’s α > 0.80) and test–retest reliability. Using a confidence level of 95% (α = 0.05), power of 0.80, expected sensitivity of 90%, and specificity of 80%, the minimum sample size was calculated to be 98 participants. The final sample (n = 105) exceeded this requirement, providing sufficient statistical power for the reliability analyses conducted in this study. The internal consistency reliability coefficients (Cronbach’s α), test–retest reliability [intraclass correlation coefficient (ICC)], and inter-rater reliability (kappa) were estimated for each subscale and the CHIRPI overall, according to the 5-point Likert scale. Correlation analysis was performed using Pearson’s test. Due to the exploratory nature of the individual questions, the statistical significance level was set at a *p*-value of less than 0.0023, based on the Bonferroni Correction for Multiple Comparisons.

## 3. Results

### 3.1. Data Screening

A total of 130 parents were approached, out of whom 110 completed the online questionnaire (response rate 84.6%). Two (2) subjects refused to participate in this research, citing that they do not know how to read or understand Greek adequately. Five of these questionnaires were excluded because the parent was a healthcare professional. No child required admission to the Pediatric Intensive Care Unit. Therefore, 105 parents participated in both phases of the questionnaire validation.

### 3.2. Participant Characteristics

Table 1 shows the basic characteristics of the participants. The majority of the sample consisted of females. Most parents were aged 31–40, married, and residing in an urban center or city (Table 1).

### 3.3. Internal Consistency

The Cronbach’s alpha reliability index, which measures the internal consistency of the CHIRPI questionnaire in Greek, was found to be 0.91 for participants in the first phase of this study (n = 105) and ranged from 0.91 to 0.97 for the individual subscales, meeting the criterion of >0.70 in all cases. In the original questionnaire, Cronbach’s alpha was found to be 0.89 during the corresponding analysis, indicating a strong mutual relationship among the items. The extraction of any question did not improve the internal consistency of the Greek version of this scale (Table 2).

### 3.4. Test–Retest Reliability

The intraclass correlation coefficient (ICC) of individual questions ranged from 0.632 to 1.000 (Table 3). This robust positive correlation indicates the presence of test–retest reliability.

### 3.5. Inter-Rater Reliability

The kappa coefficient was found to range from 0.615 to 1.00, which indicates significant to maximum reliability between the two repeated assessments of the same measure (Table 4).

Therefore, the satisfactory reliability coefficients of the scale supported the appropriate adaptation of the questionnaire for the Greek population.

### 3.6. The Results Derived from the Validation Study

The results from this sample were analyzed to explore patterns in internet-seeking behavior and their association with parents’ personal and demographic characteristics. To explore how demographic characteristics affect online behavior, we conducted subgroup analyses comparing CHIRPI responses across educational levels. Although no full correlation table was created, certain items showed statistically significant trends (Table 5). People with a high educational level were defined as those who had completed at least some higher education or who held a master’s/doctoral degree. In contrast, individuals who either had no formal education or had completed only primary school, secondary school, high school, or post-secondary education, non-university, were classified as having a lower level of education.

Notably, responses to Question 9—“After I have searched online for information about illnesses or symptoms concerning my child, I feel more anxious and distressed than before”—revealed that higher educational levels were significantly associated with increased post-search anxiety. Specifically, parents with higher education levels reported greater distress following online searches compared to those with lower educational attainment (*p* < 0.01).

Furthermore, responses to Question 11—“After searching online for health information, I ask teachers or nursery staff for their help in observing my child’s symptoms”—indicated a statistically significant trend. Most participants across all educational levels (52.4%) reported that they “1—never” seek such assistance. However, there was a tendency for individuals with higher education to do so more frequently (*p* < 0.007).

Similarly, the results of Question 5—“After I have searched online for health information, I ask teachers or nursery staff for help in implementing some of the recommendations.”—showed that the 74% of individuals belonging to some of the lower educational levels responded “1—never” to this question, while only 40% of parents with higher education responded “1—never”, and 31% of them stated that they “2—rarely” follow this practice in their daily lives (*p* < 0.004).

In Question 14—“When searching online for health information relevant to my child, I am particularly interested in possible causes for the illness”—individuals with a higher educational level (tertiary/university education or master’s/PhD degree) were more commonly interested in the identification of the possible causes of the illness during their online searches (92.7%), in contrast to individuals with a lower educational level, of whom only 60% exhibited this behavior (Pearson correlation, *p* < 0.001). The results of the remaining questions did not withstand the strict Bonferroni Correction (*p* < 0.0023).

## 4. Discussion

In the last couple of decades, the issue of parental online health information-seeking behavior has gained significant importance, while the generative AI era has added another level of complexity. This is evident from the increasing number of studies being conducted internationally. There is a lack of reliable and valid tools in Greece for assessing this specific practice and, consequently, limited knowledge regarding this type of parent behavior. Factors such as the level of health literacy, the prevalence of internet use, trust in web sources, social and familial influences, and cultural sensitivity to health issues remain unknown and warrant consideration when examining this phenomenon. Our study represents a significant step forward in understanding parental online health information-seeking behavior (e-HISB) in Greece, through the adaptation and validation of the CHIRPI questionnaire into the Greek language.

Online health information-seeking behavior (e-HISB) became increasingly prevalent and notably intensified during the COVID-19 pandemic. This practice may potentially empower individuals’ understanding of their health issues, thereby facilitating the better control of their health conditions. However, the overwhelming volume of available online health information may exacerbate feelings of fear and anxiety. Such feelings may prompt individuals to overuse medical services in search of reassurance, which, paradoxically, may heighten health-related anxiety and lead to a more negative self-assessment of their overall health [22].

Likewise, a significant number of parents utilize the internet to seek information regarding their children’s health, often leveraging this knowledge to make informed decisions. However, this search behavior may sometimes escalate into a phenomenon resembling self-directed cyberchondria [17]. On the other hand, in the era of artificial intelligence (AI), an increasing number of individuals seek health-related information by interacting with AI-based tools. A recent study based on survey data showed that ease of use, accuracy, relevance, completeness, timesaving, clarity, cost-effectiveness, and emotional coverage were significant predictors of satisfaction following conversational AI use [23]. This highlights the necessity for more comprehensive research in the field of online health information-seeking.

Despite the growing international interest in electronic health information-seeking behavior (e-HISB), particularly among parents, there is a notable lack of evidence in Greece to date. To the best of our knowledge, our study is the first attempt to adapt and validate a standardized tool in the Greek language, facilitating the identification of patterns and trends in how parents utilize online resources for health information in Greece. The Greek version of the CHIRPI questionnaire addresses this critical gap by offering a psychometrically powerful tool that is culturally and linguistically adapted for Greek-speaking populations. While no prior standardized tools or large-scale studies have been conducted in Greece in this domain, employing the CHIRPI Greek version sufficiently fulfills the relevant purpose. It enables a deeper understanding of how Greek parents interact with digital health information and is expected to support more comprehensive research in the field of online health information-seeking. While many studies have offered descriptive insights, developments in this area are still emerging [8].

In general, the issue of parental health information-seeking behavior on the internet appears to be gaining significant attention within the international scientific community. This is evidenced by the growing number of studies conducted abroad on this topic. As evidenced by the literature review conducted in the present study, there is consistency with the findings obtained from the pilot application of the tool, which was implemented as part of this research. For example, a Spanish study found that the most frequent users of the internet to find health-related information were highly educated young women. Interestingly, no significant differences were found in terms of income level, place of residence, or whether the family had children with chronic or serious illnesses [24]. Similarly, based on the limited available data regarding the Greek population, a cross-sectional study conducted in Alexandroupolis in 2021 focusing on the parents of pediatric surgical patients demonstrated that the internet is predominantly used by mothers to seek information concerning their children’s health [14]. The Greek version of CHIRPI demonstrated strong psychometric properties, including high validity and internal consistency. Regarding psychometric properties, both versions have strong internal consistency, as indicated by Cronbach’s alpha values above 0.89. Compared with the original German CHIRPI validation study, which reported an overall internal consistency of Cronbach’s α = 0.89 (Distress: 0.89; Symptom Focus: 0.87; Implementing Advice: 0.74), the Greek version demonstrated similar or even higher reliability scores (Distress: 0.97; Symptom Focus: 0.97; Implementing Advice: 0.91). This indicates that the factorial structure and internal coherence of the instrument were preserved in the Greek version, suggesting high cross-cultural equivalence. The test–retest reliability also aligns with the moderate to strong correlations found in the original study, reinforcing the scale’s stability across languages and contexts. By generating reliable and comparable data, this tool not only supports local research but also integrates Greek data into the broader international dialog on eHealth behaviors, fostering cross-cultural analysis, benchmarking, and evidence-based policymaking.

Our study has several limitations. Firstly, the cross-sectional design and the use of self-administered questionnaires limit the ability to monitor participant honesty or emotional state during completion. As a result, the accuracy of self-reported data may be affected. Secondly, the sample consisted of parents whose children were hospitalized in a single pediatric department in a university hospital in Greece, which may limit the generalizability of the findings to the broader population of Greek parents or those in other healthcare settings. The use of a non-random, convenience sampling approach may also introduce selection bias. In addition, the low participation rate of fathers is a noteworthy limitation, as mothers comprised the majority of respondents. One significant limitation of our study is that the validity assessment focused only on internal consistency and test–retest reliability. We did not conduct convergent validity analyses with related instruments, such as the eHEALS or CSS. This oversight represents a limitation in establishing the construct validity of CHIRPI. Finally, this study did not conduct structural validity analysis (e.g., exploratory factor analysis) or sensitivity testing and relied exclusively on self-reported data, which may be subject to bias.

Future studies should aim to explore parental online health-seeking behaviors in non-hospitalized contexts, where health-related anxiety might be lower and decision-making dynamics may differ significantly. In this way, although the CHIRPI Greek version demonstrated strong psychometric properties in terms of internal consistency and test–retest reliability, further validation is needed in diverse populations, including those from different geographic regions, genders, age ranges, socioeconomic backgrounds, and educational levels. Although our findings are promising, the absence of factor analysis and sensitivity testing warrants careful interpretation. Further validation in larger and more diverse samples is essential.

## 5. Conclusions

In conclusion, we developed a valid and reliable Greek-language version of the Children’s Health Internet Research, Parental Inventory (CHIRPI) tool. As the first standardized instrument in Greek designed to assess excessive parental online health information-seeking regarding children’s health, CHIRPI addresses a significant gap in eHealth assessment within the Greek-speaking population. Its strong psychometric properties confirm its suitability for use in research contexts. This tool will serve as a foundation for future research in parental digital health literacy, assist health professionals in understanding culturally specific patterns of information-seeking behaviors, and guide policymakers in designing targeted interventions. Moreover, its availability paves the way for cross-cultural comparisons and benchmarking with international data on eHealth behaviors, ultimately contributing to the development of effective and responsible digital health strategies in pediatric care.

## Figures and Tables

**Table 1:** Participant demographic characteristics.

		n	%
**Gender**	Male	35	33.33
	Female	70	66.67
**Age Group**	Up to 24 years old	1	0.95
	25–30 years old	17	16.19
	31–40 years old	49	46.67
	41–50 years old	34	32.38
	Over 51 years old	4	3.81
**Marital Status**	Married	101	96.19
	Single	1	0.95
	Divorced	3	2.86
	Widowed	0	0.00
**Educational Level**	No formal education	5	4.76
	Primary school	0	0.00
	Middle school	0	0.00
	High school	28	26.67
	Post-secondary education (craft training)	17	16.19
	Tertiary education (college, university)	39	37.14
	Postgraduate/master’s/doctorate	16	15.24
**Place of Residence**	Urban center/city	80	76.19
	Rural area/village	25	23.81
**Chronic Disease in Family**	None	85	80.95
	Mother	9	8.57
	Father	2	1.90
	Child aged 0–10 years	9	8.57
	Sibling (older)	2	1.90

Values are expressed as absolute numbers (n) and percentages (%).

**Table 2:** Internal consistency reliability—Cronbach’s alpha.

	Translated Scale	Official Scale
	Cronbach’s Alpha	Standardized Alpha
**Distress**	0.97	0.89
**Symptom focus**	0.97	0.87
**Implementing advice**	0.91	0.74
**CHIRPI (total scale)**	0.91	0.89

**Table 3:** Test–retest reliability—test–retest intraclass correlation coefficient.

	Test–Retest ICC Intraclass Correlation Coefficient	Lower Bound–Upper Bound
Question 1 (1a–1b)	0.858	0.798–0.901
Question 2 (2a–2b)	1.000	1.000–1.000
Question 3 (3a–3b)	0.705	0.594–0.789
Question 4 (4a–4b)	0.862	0.803–0.904
Question 5 (5a–5b)	0.632	0.502–0.735
Question 6 (6a–6b)	0.899	0.855–0.930
Question 7 (7a–7b)	0.862	0.804–0.904
Question 8 (8a–8b)	0.778	0.690–0.844
Question 9 (9a–9b)	0.920	0.884–0.945
Question 10 (10a–10b)	0.884	0.834–0.920
Question 11 (11a–11b)	0.865	0.807–0.906
Question 12 (12a–12b)	0.751	0.653–0.823
Question 13 (13a–13b)	0.657	0.533–0.753
Question 14 (14a–14b)	0.888	0.840–0.923
Question 15 (15a–15b)	0.853	0.791–0.898
Question 16 (16a–16b)	0.839	0.772–0.888
Question 17 (17a–17b)	0.817	0.742–0.872
Question 18 (18a–18b)	0.885	0.836–0.921
Question 19 (19a–19b)	0.797	0.714–0.857
Question 20 (20a–20b)	0.759	0.664–0.830
Question 21 (21a–21b)	0.882	0.831–0.918

a: Responses from the completion of the 1st questionnaire. b: Responses from the completion of the 2nd questionnaire.

**Table 4:** Inter-rater reliability—kappa.

	Kappa
Question 1 (1a–1b)	0.743
Question 2 (2a–2b)	1.000
Question 3 (3a–3b)	0.649
Question 4 (4a–4b)	0.685
Question 5 (5a–5b)	0.615
Question 6 (6a–6b)	0.717
Question 7 (7a–7b)	0.680
Question 8 (8a–8b)	0.734
Question 9 (9a–9b)	0.791
Question 10 (10a–10b)	0.757
Question 11 (11a–11b)	0.726
Question 12 (12a–12b)	0.704
Question 13 (13a–13b)	0.711
Question 14 (14a–14b)	0.713
Question 15 (15a–15b)	0.684
Question 16 (16a–16b)	0.878
Question 17 (17a–17b)	0.703
Question 18 (18a–18b)	0.662
Question 19 (19a–19b)	0.654
Question 20 (20a–20b)	0.757
Question 21 (21a–21b)	0.744

a: Responses from the completion of the 1st questionnaire. b: Responses from the completion of the 2nd questionnaire.

**Table 5:** Educational differences in CHIRPI responses.

Item (CHIRPI Question)	Behavior Assessed	Educational Group	% Reporting Behavior	*p*-Value
Q9: “After I have searched online for information about illnesses or symptoms concerning my child, I feel more anxious and distressed than before.”	Distress	Tertiary/Postgraduate	11% (vs. 38%) reported “never”	<0.01
Q11: “After searching online for health information, I ask teachers or nursery staff for their help in observing my child’s symptoms.”	Implementing Advice	All	52.4% reported “never”	<0.007
Q5: “After I have searched online for health information, I ask teachers or nursery staff for help in implementing some of the recommendations.”	Implementing Advice	Low Levels	74% reported “never”	<0.004
Q14: “When searching online for health information relevant to my child, I am particularly interested in possible causes for the illness.”	Symptom Focus	High Levels	92.7% (vs. 60%) reported yes, even if “rarely”	<0.001

## Data Availability

The data that support the findings of this study are not publicly available due to ethical or privacy restrictions. Interested parties may contact the corresponding author for further information, subject to institutional approvals.

## Supplementary Materials

The Greek translation of CHIRPI is available online free of charge as the CHIRPI Greek version at https://elocus.lib.uoc.gr/dlib/a/6/c/metadata-dlib-1682596458-173188-14367.tkl (accessed on 26 June 2025).

## Abbreviations

The following abbreviations are used in this manuscript:

e-HISB	Electronic health information-seeking behavior
HRI	Health-related information
CHIRPI	Children’s Health Internet Research, Parental Inventory
ΔAΠΥΓΚ	Διαδικτυακή Aναζήτηση Παιδικής Υγείας, Γονική Καταγραφή

## Appendix A

Presentation of the various stages involved in the translation process of the CHIPRI questionnaire.

## References

[1] Brashers D.E., Goldsmith D.J., Hsieh E. (2002). Information Seeking and Avoiding in Health Contexts. Hum. Commun. Res..

[2] Rains S.A. (2007). Perceptions of Traditional Information Sources and Use of the World Wide Web to Seek Health Information: Findings from the Health Information National Trends Survey. J. Health Commun..

[3] Napoli P.M. (2001). Consumer Use of Medical Information from Electronic and Paper Media: A Literature Review. The Internet and Health Communication: Experiences and Expectations.

[4] AlGhamdi K.M., Moussa N.A. (2012). Internet use by the public to search for health-related information. Int. J. Med. Inform..

[5] Semere W., Karamanoukian H.L., Levitt M., Edwards T., Murero M., D’Ancona G., Donias H.W., Glick P.L. (2003). A pediatric surgery study: Parent usage of the internet for medical information. J. Pediatr. Surg..

[6] Frey E., Bonfiglioli C., Brunner M., Frawley J. (2022). Parents’ Use of Social Media as a Health Information Source for Their Children: A Scoping Review. Acad. Pediatr..

[7] Sebelefsky C., Voitl J., Karner D., Klein F., Voitl P., Böck A. (2016). Internet use of parents before attending a general pediatric outpatient clinic: Does it change their information level and assessment of acute diseases?. BMC Pediatr..

[8] Kubb C., Foran H.M. (2020). Online Health Information Seeking by Parents for Their Children: Systematic Review and Agenda for Further Research. J. Med. Internet Res..

[9] Holyoake D.-D., Searle K. (2015). Cyberchondria: Emerging themes for children’s nurses in the internet age. Nurs. Child. Young People.

[10] Xiang H., Zhou J., Liu M., Gao Q., Zhou J. (2023). Judging Online Health Misinformation: Effects of Cyberchondria and Age. Human Aspects of IT for the Aged Population.

[11] Norman C.D., Skinner H.A. (2006). eHEALS: The eHealth Literacy Scale. J. Med. Internet Res..

[12] McElroy E., Kearney M., Touhey J., Evans J., Cooke Y., Shevlin M. (2019). The CSS-12: Development and Validation of a Short-Form Version of the Cyberchondria Severity Scale. Cyberpsychol. Behav. Soc. Netw..

[13] Yardi S., Caldwell P.H., Barnes E.H., Scott K.M. (2018). Determining parents’ patterns of behaviour when searching for online information on their child’s health. J. Paediatr. Child Health.

[14] Aggelidou M., Deftereos S.P., Cassimos D.C., Skarentzos K., Oikonomou P., Angelidou A., Nikolaou C., Koufopoulos G., Kambouri K. (2021). Influence of education and residence on the parental search for pediatric surgical information on the internet. World J. Clin. Pediatr..

[15] Nicholl H., Tracey C., Begley T., King C., Lynch A.M. (2017). Internet Use by Parents of Children with Rare Conditions: Findings from a Study on Parents’ Web Information Needs. J. Med. Internet Res..

[16] Jaks R., Baumann I., Juvalta S., Dratva J. (2019). Parental digital health information seeking behavior in Switzerland: A cross-sectional study. BMC Public Health.

[17] Barke A., Doering B.K. (2020). Development of an Instrument to Assess Parents’ Excessive Web-Based Searches for Information Pertaining to Their Children’s Health: The “Children’s Health Internet Research, Parental Inventory” (CHIRPI). J. Med. Internet Res..

[18] World Health Organization (2023). Global Scales for Early Development (GSED) Package: Version 1.0.

[19] Perneger T.V., Courvoisier D.S., Hudelson P.M., Gayet-Ageron A. (2015). Sample size for pre-tests of questionnaires. Qual. Life Res. Int. J. Qual. Life Asp. Treat. Care Rehabil..

[20] Tsang S., Royse C.F., Terkawi A.S. (2017). Guidelines for developing, translating, and validating a questionnaire in perioperative and pain medicine. Saudi J. Anaesth..

[21] Higgins P.A., Straub A.J. (2006). Understanding the error of our ways: Mapping the concepts of validity and reliability. Nurs. Outlook.

[22] Di Novi C., Kovacic M., Orso C.E. (2024). Online health information seeking behavior, healthcare access, and health status during exceptional times. J. Econ. Behav. Organ..

[23] Esmaeilzadeh P., Maddah M., Mirzaei T. (2025). Using AI chatbots (e.g., CHATGPT) in seeking health-related information online: The case of a common ailment. Comput. Hum. Behav. Artif. Hum..

[24] Nievas-Soriano B.J., Castro-Luna G.M., García-Duarte S., González-López M.D.C., Parrón-Carreño T. (2021). Profile of the Users and the Most Visited Topics of a Pediatric eHealth Website. Int. J. Environ. Res. Public Health.

